# Effect of switching from tenofovir disoproxil fumarate to tenofovir alafenamide on lipid profiles in patients with hepatitis B

**DOI:** 10.1371/journal.pone.0261760

**Published:** 2022-01-20

**Authors:** Kazuharu Suzuki, Goki Suda, Yoshiya Yamamoto, Satoshi Abiko, Kenji Kinoshita, Shuichi Miyamoto, Ryo Sugiura, Megumi Kimura, Osamu Maehara, Ren Yamada, Takashi Kitagataya, Taku Shigesawa, Masatsugu Ohara, Naoki Kawagishi, Masato Nakai, Takuya Sho, Mitsuteru Natsuizaka, Kenichi Morikawa, Koji Ogawa, Naoya Sakamoto

**Affiliations:** 1 Department of Gastroenterology and Hepatology, Graduate School of Medicine, Hokkaido University, Hokkaido, Japan; 2 Department of Gastroenterology, Hakodate Municipal Hospital, Hokkaido, Japan; Centre de Recherche en Cancerologie de Lyon, FRANCE

## Abstract

For long-term treatment of hepatitis B virus (HBV) infection, switching from tenofovir-disoproxil-fumarate (TDF) to tenofovir-alafenamide (TAF) may prevent renal dysfunction and bone loss. However, the precise effects of this switch on the blood lipid profile remain to be clarified. This is an important issue as TDF is known to have effects on both low- and high-density lipids. Therefore, our retrospective multi-center study aimed to evaluate the effects of switching from TDF to TAF on the lipid profile of patients with HBV infection. Samples were obtained prior to the switch from TDF to TAF and at 6–12 months after TAF initiation. In some cases, additional samples obtained pre- and post-TDF administration were available for analysis. Serum cholesterol levels, including oxidized-low-density lipoprotein (LDL) and non-high-density lipoprotein-cholesterol (HDL-c), and the rate of dyslipidemia, according to the NCEP-ATP III lipid risk classification, were analyzed. The data from 69 patients were analyzed, including 33 patients with pre- and post-TDF-initiation serum samples. Total cholesterol (T-chol), HDL-c, LDL-c, non-HDL-c, and oxidized LDL levels increased significantly after switching to TAF. With regard to sequential changes pre- to post-TAF, TDF was associated with significantly lower serum T-chol, HDL-c, and oxidized LDL-c levels, with T-chol, HDL-c, LDL-c, and oxidized LDL-c levels increasing significantly after the switch. The switch from TDF to TAF was also associated with an increase in the rate of dyslipidemia, from 33% to 39%, with an increase in the rate of severe dyslipidemia of 1.4% and 5.8%, based on T-chol and LDL-c levels. Of note, no cases of severe dyslipidemia were detected pre-TAF treatment. As oxidized LDL-c and non-HDL-c are strongly associated with atherosclerosis development, careful monitoring of lipid is needed after switching from TDF to TAF in this clinical population.

## Introduction

Hepatitis B virus (HBV) infection is a major cause of hepatocellular carcinoma (HCC) and liver cirrhosis and accounts for approximately 1 million worldwide deaths annually [1–3]. Thus, effective therapeutic strategies to manage HBV infection are needed. Currently, interferon and nucleos(t)ide analogs (NAs) are the most common therapies used for patients with an HBV infection, with good effectiveness and safety profiles having been achieved. However, NAs can require consecutive, and even indefinite, cycles of administration [4]. Lamivudine, adefovir dipivoxil, entecavir (ETV), tenofovir-disoproxil-fumarate (TDF), and tenofovir alafenamide (TAF) are approved NAs for chronic hepatitis B and are considered as first-line NAs for HBV infection [4–7]. Both TDF and TAF are pro-drugs of tenofovir [8]. However, long-term administration of TDF can cause renal dysfunction and bone loss [9–11]. By contrast, TAF was designed to have greater stability than TDF, such that the active metabolite of tenofovir diphosphate is delivered to the liver more efficiently. As such, the required dose of TAF (25 mg once a day) is lower than the required dose of TDF (300 mg once a day), resulting in less kidney and bone toxicity [8]. Switching from TDF to TAF has been conducted in general clinical practice for patients who are at risk of renal dysfunction and bone loss.

The positive intra- and extrahepatic effects of TDF are important to note, however, when considering a switch from TDF to TAF. Murata et al. [12] clearly demonstrated that TDF promotes the release of interferon-λ3 by gastrointestinal cells, which could affect serum hepatitis B surface antigen (HBsAg) levels. In addition, we and other groups have shown that, in patients with HBV and/or human immunodeficiency virus (HIV) infection, TDF reduces the serum cholesterol level through the upregulation of the CD36/PPAR-alpha axis [13–15]. Previous research has shown that switching from TDF to TAF increases serum cholesterol levels in patients with HIV infection [16]. However, it is important to note that TDF may increase both low-density lipoprotein cholesterol (LDL-c) and high-density lipoprotein cholesterol (HDL-c) levels, wherein an increase in LDL-c can promote development of atherosclerosis, while an increase in HDL-c can provide a protective effect [17]. Thus, the actual effect of switching from TDF to TAF on the development of atherosclerosis is unclear.

We have recently reported that TDF could decrease serum oxidized LDL-c [15], which is strongly associated with the development of atherosclerosis [18, 19]. However, the kinetics of oxidized LDL-c after switching from TDF to TAF has not yet been clearly established. Therefore, this study aimed to evaluate the effect of switching from TDF to TAF on the lipid profile of patients with a chronic HBV infection. Additionally, we analyzed sequential changes in lipid profile components, including oxidized LDL-c, from baseline to TDF administration, after switching from TDF to TAF, and post-TAF administration for patients who had proper clinical information and preserved serum samples.

## Methods

### Patients and clinical study design

This was a retrospective multicenter study of patients with chronic hepatitis B who switched from TDF to TAF, recruited between 2016 and 2020 from hospitals in the NORTE Study Group, who performed clinical studies of liver diseases [20–25]. Included were patients with an HBV infection who were ≥20 years of age, had switched from TDF to TAF for the management of their HBV infection and received TAF for >6 months, and for whom the required information was available in their medical record and preserved serum samples at baseline and 6–12 months after the initiation of TAF were available. Patients with additional clinical information and preserved serum samples at all time points (pre-TDF administration, post-TDF administration [6–12 months], pre-TAF administration, and post-TAF administration [6–12 months]) were included in detailed analyses of sequential changes in the lipid profile. Patients were excluded if they had a history of liver disease other than chronic hepatitis B, uncontrolled malignancy, including HCC, a history of treatment for dyslipidemia, had received prophylactic NA for HBV reactivation, or declined to participate in the study.

Clinical information was collected, including laboratory data, HBV infectious status, and concomitant drug information, in addition to preserved serum samples. Changes in cholesterol levels were analyzed in preserved serum samples. TAF (25 mg) or TDF (300 mg) was administered once daily according to the manufacturer’s recommendation.

### Statement of ethics

The study protocol conformed to the ethical guidelines of the Declaration of Helsinki and was approved by the ethics committee of Hokkaido University Hospital (Clinical research number: IRB 017–0387). All enrolled patients provided written informed consent to participate in this study or did not decline to participate in this study. All data were fully anonymized before we accessed them, and the ethics committee specifically approved non-decline of being included in the study in lieu of written informed consent for some patients.

### Serum HBV markers and lipid profile analysis

Serum total cholesterol (T-chol), HDL-c, LDL-c, and oxidized LDL levels were analyzed by enzyme-linked immunosorbent assays (SRL, Tokyo, Japan), according to manufacturer’s instructions, as previously described [15]. Serum HBsAg and HBV-DNA levels were analyzed by chemiluminescence immunoassay (Architect HBsAg-QT assay; Abbott Laboratory, Tokyo, Japan) and a real-time TaqMan PCR assay (Cobas AmpliPrep/Cobas TaqMan HBV Test, v2.0) [10, 26]. The change in the rate of dyslipidemia was evaluated based on the National Cholesterol Education Program-Adult Panel III (NCEP-ATP III) lipid risk classification [27].

### Statistical analysis

Categorical variables were analyzed using the *χ*2 test. Continuous variables were analyzed using the paired *t-*test or Mann–Whitney *U* test. Differences among three or more groups were evaluated using Friedman’s test and Dunn’s multiple comparisons tests. Differences in the proportion of NCEP-ATP III classification-based dyslipidemia between pre-TAF and post-TAF samples were assessed using the extended McNemar’s test. To analyze changes in the rate of dyslipidemia across time points, multiple logistic regression was used. All P-values were two-tailed, and the level of significance was set at P < 0.05. Statistical analyses were conducted using Prism 7.03 (GraphPad Software, Inc., La Jolla, CA, USA).

## Results

### Patient characteristics

**Fig 1:**
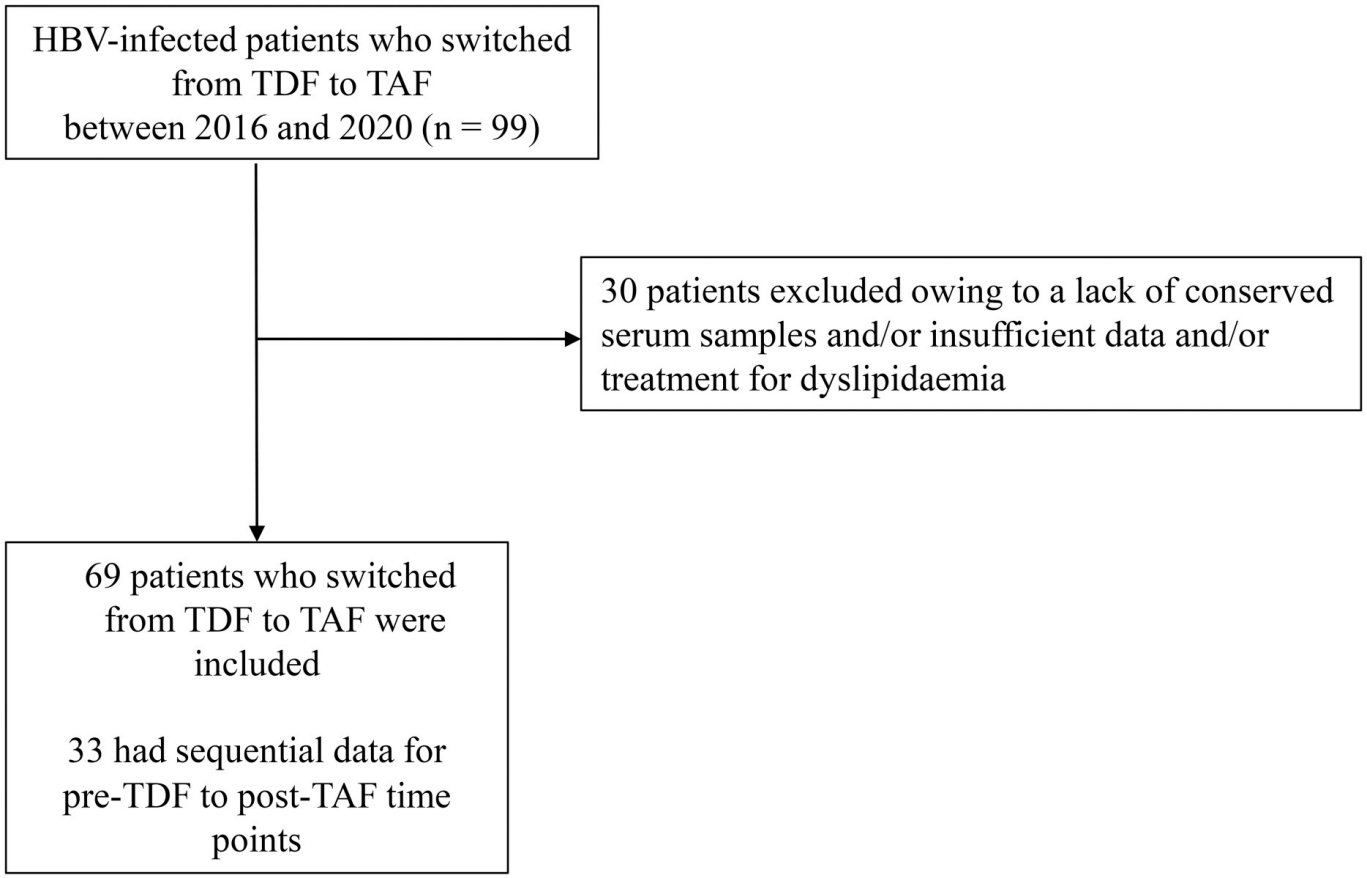
Study flow. TDF, tenofovir-disoproxil-fumarate; TAF, tenofovir alafenamide.

A total of 99 patients with chronic hepatitis B were switched from TDF to TAF between 2016 and 2020. Among these, 30 patients were excluded owing to a lack of preserved serum samples, insufficient data, and/or treatment for dyslipidemia (Fig 1), with the remaining 69 patients enrolled (Table 1). Preserved serum samples obtained pre-TDF initiation and post-TDF administration (6–12 months after TDF initiation) were available from 33 of these 69 patients for analyses of sequential changes in lipid profiles and clinical data (Table 1).

**Table 1:** Baseline characteristics of patients before TAF treatment.

Total number	69
**Age (years)** ^a^	61 (27–83)
**Age (years) >70 n (%)**	14 (20.3%)
**Sex (male/female)**	45/24
**Body mass index (kg/m** ^**2**^ **)**	23.2 (17.4–33.2)
**Body mass index >25 n (%)**	20 (37.7%)
**Chronic hepatitis/liver cirrhosis**	55/14
**Baseline platelet count (×10**^**4**^**/μL)** ^a^	17.9 (3.2–33.1)
**Alb (g/dL)** ^a^	4.4 (3.1–4.8)
**Baseline AST level (IU/L)** ^a^	25 (15–79)
**Baseline ALT level (IU/L)** ^a^	22 (6–157)
**Baseline γGT (IU/L)** ^a^	26 (10–296)
**Baseline AFP (ng/mL)** ^a^	2.7 (1.3–20.9)
**Baseline creatinine (mg/dL)** ^a^	0.91 (0.54–2.56)
**eGFR (mL/min/1.73 m**^**2**^**)** ^a^	64.5 (20.9–135.0)
**FIB-4 index** ^a^	1.69 (0.57–20.06)
**FIB-4 index >3.25 n (%)**	11 (15.9%)
**History of malignancy n (%)**	17 (24.6%)
**Genotype (B/C/unknown)** ^a^	10/37/22
**HBs antigen (IU/mL)** ^a^	893.1 (0.2–33298.2)
**HBe antigen-positive (%)**	17 (30.4%)
**HBV-DNA (Log copies/mL)** ^a^	2.1 (2.1–3.5)
**Baseline lipid profile**	
**T-chol (mg/dL)** ^a^	180 (124–238)
**HDL-c (mg/dL)** ^a^	52 (17–116)
**LDL-c (mg/dL)** ^a^	93 (43–145)
**Oxidized LDL (U/L)** ^a^	101 (39–228)
**Non-HDL-c (mg/dL)** ^a^	127 (66–193)

Alb, albumin; AST, aspartate aminotransferase; ALT, alanine transaminase; γGT, gamma-glutamyl transferase; eGFR, estimated glomerular filtration rate; HCC, hepatocellular carcinoma, HBs, hepatitis B surface; HBe, hepatitis B envelope; HBV-DNA, hepatitis B virus-deoxyribonucleic acid; LDL-c, low-density lipoprotein cholesterol; AFP, alpha fetoprotein.

^a^ Data are shown as median (range).

### Lipid profile changes after switching from TDF to TAF in patients with chronic hepatitis B

**Fig 2:**
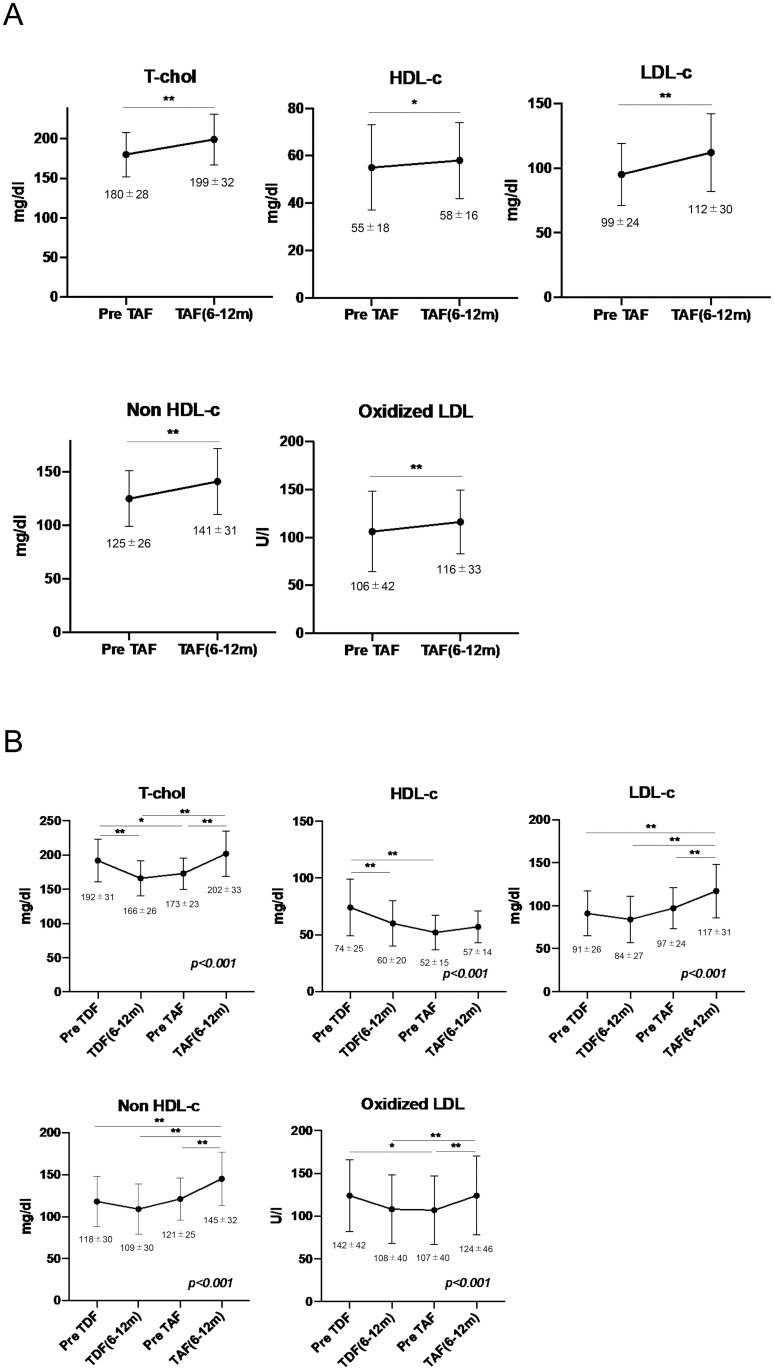
Changes in cholesterols after switching from TDF to TAF. A. Changes in T-chol, HDL-c, LDL-c, non-HDL-c, and oxidized LDL-c from pre-TAF to post-TAF administration (6–12 months); n = 69. B. Differences in T-chol, HDL-c, LDL-c, non-HDL-c, and oxidized LDL-c among pre-TDF administration, post-TDF administration, pre-TAF administration, and post-TAF administration (6–12 months); n = 33. The duration of TDF administration was 29.1 ± 8.4 months. TDF, tenofovir-disoproxil-fumarate; TAF, tenofovir alafenamide; T-chol, total cholesterol; HDL-c, high-density lipoprotein cholesterol; LDL-c, low-density lipoprotein cholesterol. Data are shown as means ± standard deviation for triplicate assays. *P < 0.05 or **P < 0.01.

As shown in Fig 2A, the switch from TDF to TAF significantly increases the levels of T-chol (P < 0.01), HDL-c (P < 0.05), LDL-c (P < 0.01), non-HDL-c (P < 0.01), and oxidized LDL (P< 0.01) at 6–12 months after the initiation of TAF. Similar tendencies were observed both in patients with liver cirrhosis and chronic hepatitis (S1 Fig). The sequential analysis of samples from the 33 patients with sufficient serum samples revealed that TDF administration significantly lowered serum T-chol, HDL-c, and oxidized LDL-c levels. The switch from TDF to TAF significantly increased T-chol, LDL-c, and oxidized LDL-c levels (Fig 2B).

### Changes in the rate of dyslipidemia after switching from TDF to TAF

**Fig 3:**
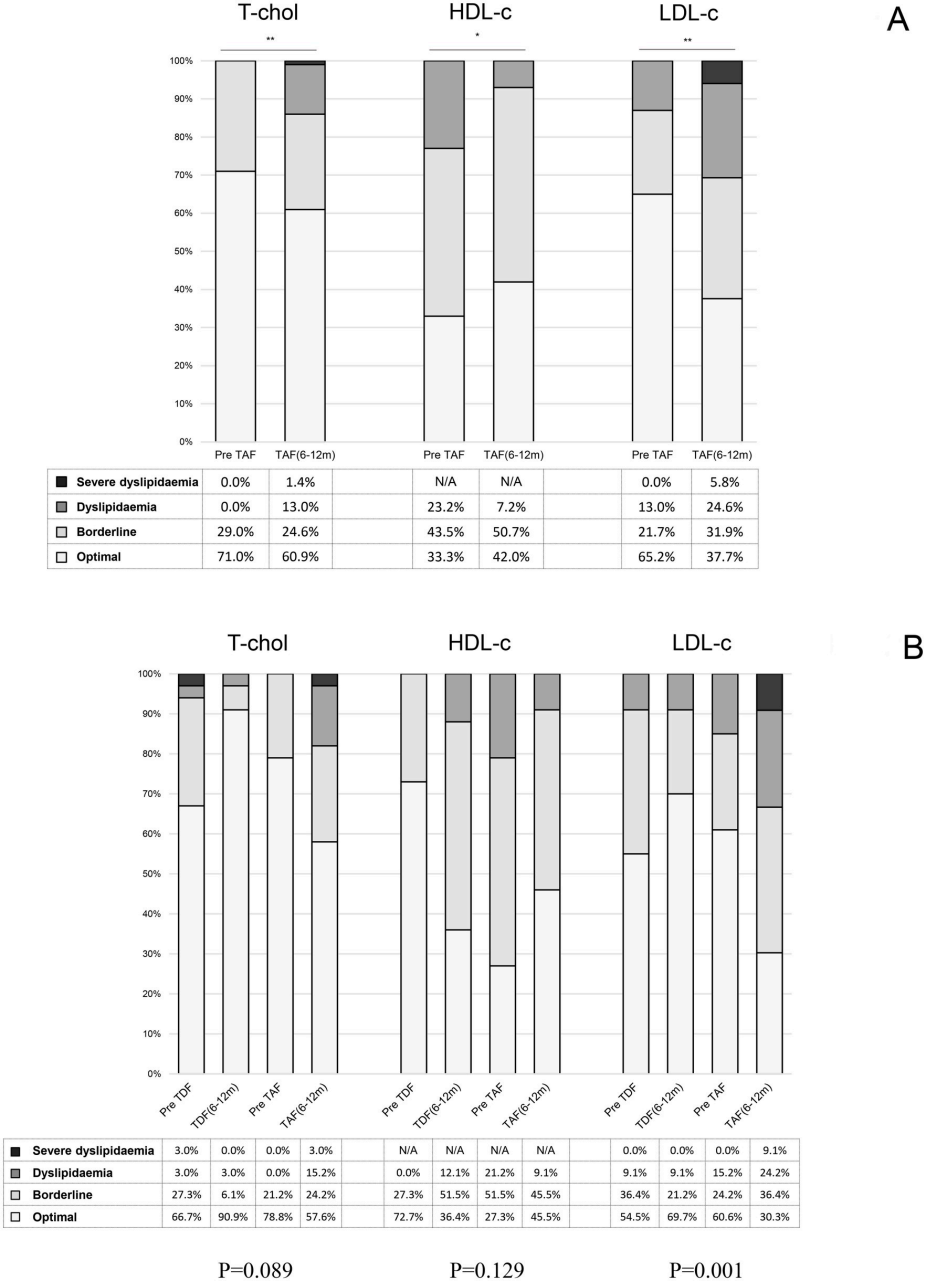
Changes in the rate of dyslipidemia according to the NCEP-ATP III lipid risk classification after switching from TDF to TAF. A. Changes in the rate of dyslipidemia according to the NCEP-ATP III lipid risk classification between pre-TAF administration and post-TAF administration (6–12 months); n = 69. The change in the proportion of patients with NCEP-ATP III classification-based dyslipidemia between pre-TAF and post-TAF was assessed using the extended McNemar’s test. B. Differences in the rate of dyslipidemia according to the NCEP-ATP III classification among pre-TDF administration, post-TDF administration, pre-TAF administration, and post-TAF administration (6–12 months) time points; n = 33. To analyze associations between treatment points and the rate of dyslipidemia, multiple logistic regression was performed. TDF, tenofovir-disoproxil-fumarate; TAF, tenofovir alafenamide. Data are shown as means ± standard deviation for triplicate assays. *P < 0.05 or **P < 0.01.

We analyzed the change in the proportion of patients with dyslipidemia according to the NCEP-ATP III lipid risk classification before and after the switch from TDF to TAF. As shown in Fig 3A, the rate of dyslipidemia, as evaluated by T-chol and LDL-C, increased significantly after switching from TDF to TAF. The rate of dyslipidemia, as determined by HDL-c, decreased significantly after switching from TDF to TAF. Overall, the proportion of patients with dyslipidemia based on any indicator increased from 33% to 39% by switching from TDF to TAF. In addition, the rate of severe dyslipidemia increased to 1.4% to 5.8%, based on T-chol and LDL-c levels, respectively, after the switch from TDF to TAF, with no case of severe dyslipidemia identified pre-TAF treatment. Furthermore, the sequential analysis indicated a significant difference in the proportion of patients with dyslipidemia (severe dyslipidemia plus dyslipidemia), based on the LDL-c level and the NCEP-ATP III lipid risk classification, from pre-TDF to post-TDF (6–12 months) administration (P = 0.001; Fig 3B).

### Changes in renal function, aspartate aminotransferase (AST), alanine aminotransferase (ALT), and HBV infection status after switching from TDF to TAF

**Fig 4:**
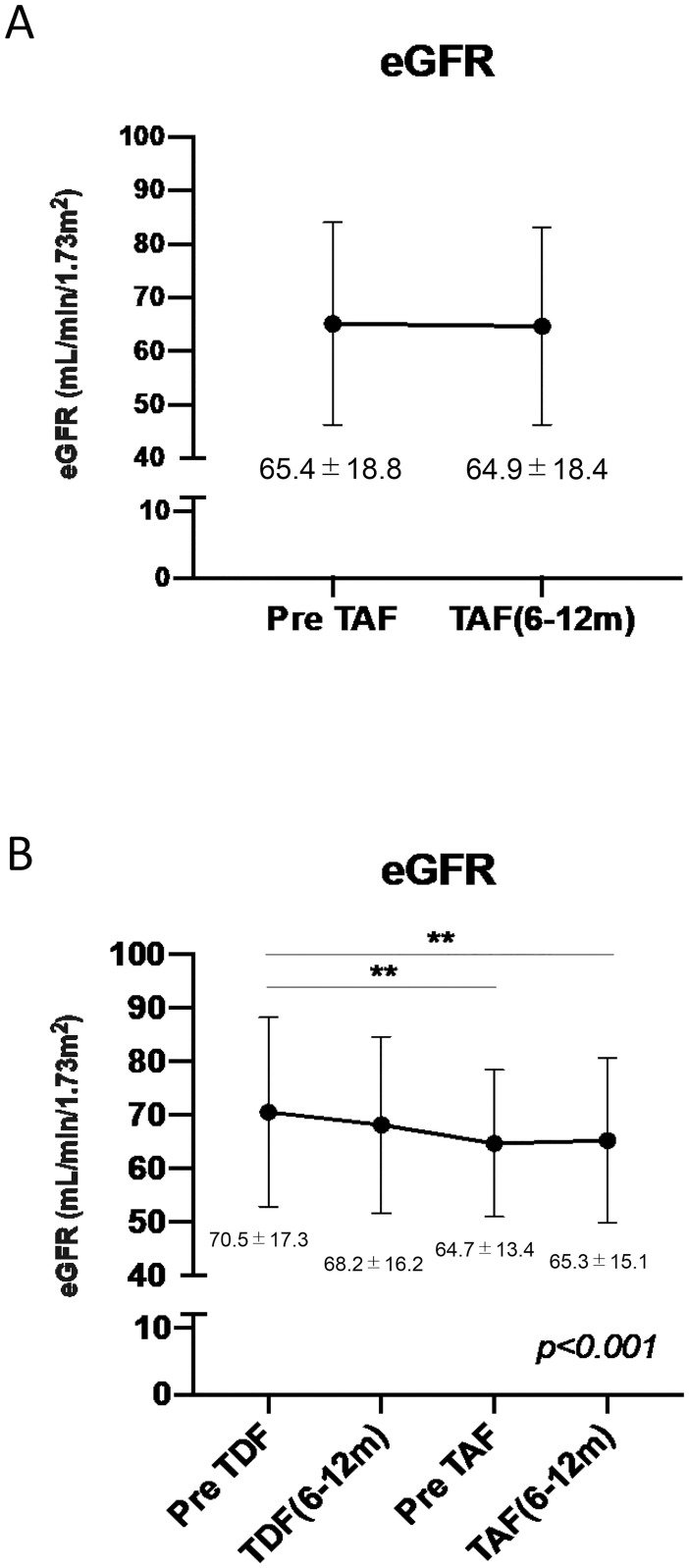
Changes in the eGFR after switching from TDF to TAF. A. Changes in eGFR between pre-TAF administration and post-TAF administration (6–12 months); n = 69. B. Differences in eGFR among pre-TDF administration, post-TDF administration, pre-TAF administration, and post-TAF administration (6–12 months) time points; n = 33. TDF, tenofovir-disoproxil-fumarate; TAF, tenofovir alafenamide; eGFR, estimated glomerular filtration rate. Data are shown as means ± standard deviation for triplicate assays. *P < 0.05 or **P < 0.01.

**Fig 5:**
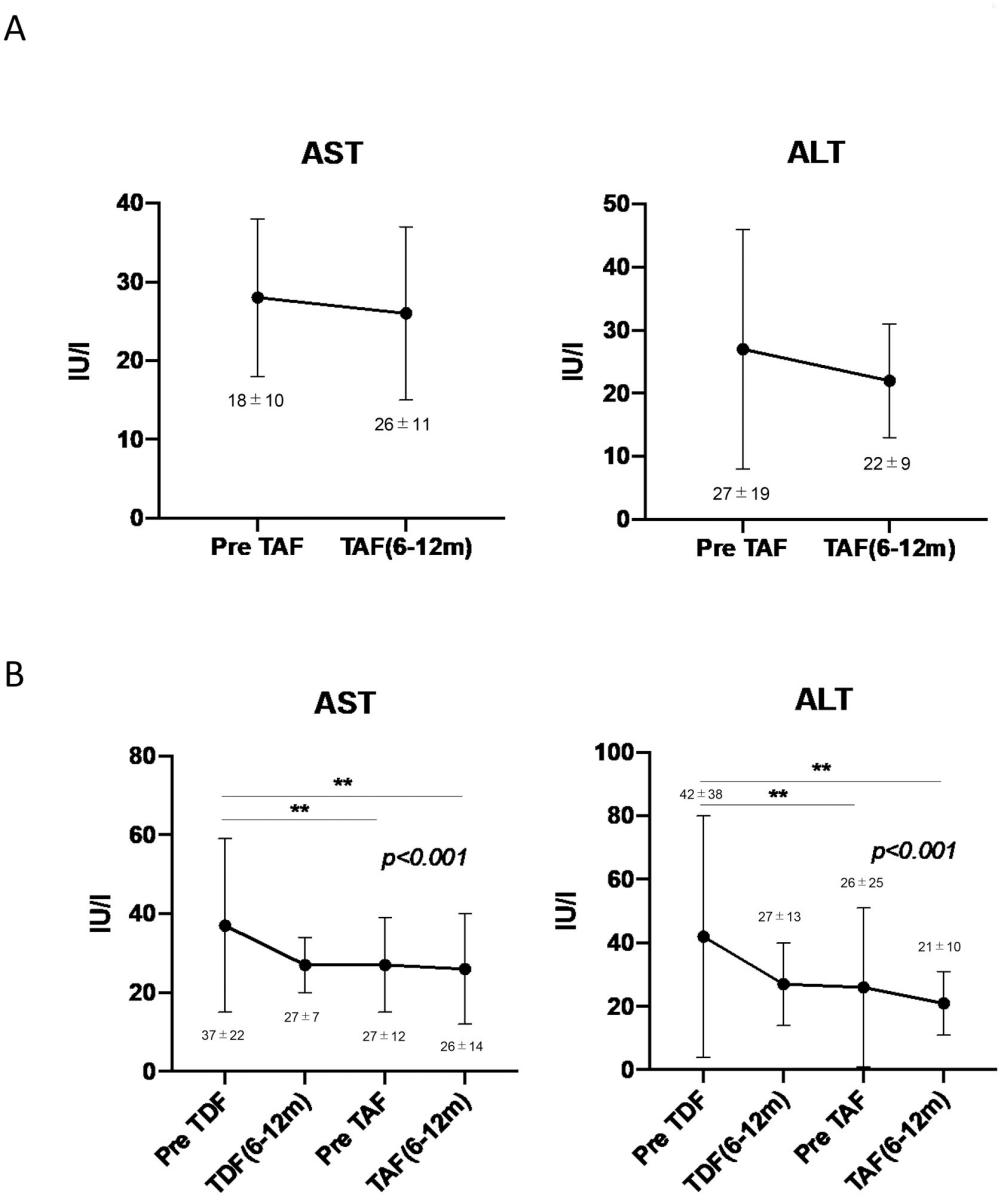
Changes in AST and ALT levels after switching from TDF to TAF. A. Changes in AST and ALT levels between pre-TAF administration and post-TAF administration (6–12 months); n = 69. B. Changes in AST and ALT levels among pre-TDF administration, post-TDF administration, pre-TAF administration, and post-TAF administration (6–12 months); n = 33. TDF, tenofovir-disoproxil-fumarate; TAF, tenofovir alafenamide; AST, aspartate aminotransferase; ALT, alanine aminotransferase. Data are shown as means ± standard deviation for triplicate assays. *P < 0.05 or **P < 0.01.

As shown in Figs 4A and 5A, after switching from TDF to TAF, the estimated glomerular filtration rate (eGFR), AST, and ALT levels at 6–12 months after TAF initiation and those prior to TAF administration were not different. Among the 33 patients included in the sequential analysis, the mean eGFR, AST, and ALT levels decreased significantly from pre- to post-TDF administration and were not affected by switching from TDF to TAF (Figs 4B and 5B). As shown in Table 2, HBsAg levels decreased significantly with both TDF and TAF administration.

**Table 2:** Effects of switching from TDF to TAF on HBV-DNA and HBs antigen levels.

	**Pre-TAF**	**TAF (6–12 m)**	**P-value**	
**HBs antigen (IU/mL)** ^a^	893.1 (0.2–33298.2)	629.0 (0.1–26166.1)	**0.007**	
**HBV-DNA (Log copies/mL)** ^a^	2.1 (2.1–3.5)	2.1 (2.1–3.6)	0.927	
	**Pre-TDF**	**Pre-TAF**	**TAF (6–12 m)**	**P-value**
**HBs antigen (IU/mL)** ^a^	2077.1 (4.8–115844.0)	1031.1 (2.1–33298.2)	630.5 (0.7–26166.1)	**<0.001**
**HBV-DNA (Log copies/mL)** ^a^	4.8 (2.1–9.0)	2.1 (2.1–2.9)	2.1 (2.1–2.6)	**<0.001**

HBs, hepatitis B surface; HBV-DNA, hepatitis B virus-deoxyribonucleic acid.

^a^ Data are shown as median (range).

## Discussion

Our results indicate that switching from TDF to TAF significantly increased T-chol, LDL-c, HDL-c, oxidized LDL-c, non-HDL-c levels, as well as the rate of dyslipidemia, in patients with chronic hepatitis B. As oxidized LDL-c and non-HDL-c levels are strongly associated with the development of atherosclerosis [18, 19, 28], careful monitoring of these parameters is needed when switching from TDF to TAF.

NAs are safe and effective for patients with HBV infection and thus are widely used, even for long-term treatment. In particular, ETV, TDF, and TAF are first-line NAs for the management of HBV infection [5]. However, long-term TDF administration is associated with renal dysfunction and bone loss [9–11]. For the treatment of HBV infection over a 48-week period, the effect of TAF is non-inferior to that of TDF, with improved bone and renal safety compared with that of TDF [29]. Over an extended duration of 96 weeks, TAF is as effective as TDF, with continued improved renal and bone safety [8]. Thus, patients with a risk of renal and bone diseases may benefit from switching from TDF to ETV or TAF, according to the European Association for the Study of the Liver guideline [5]. In our study, eGFR did not change after switching from TDF to TAF (Fig 4A) although gradually declined during TDF treatment (Fig 4B).

In patients with HIV and/or HBV infection, previous reports have shown that TDF administration lowers T-Chol, LDL-c, and HDL-c levels simultaneously [13–15]. Consistent with these previous reports, in our study, cholesterol levels decreased significantly during TDF administration (Fig 2B). However, other reports have shown that switching from TDF to TAF increased levels of cholesterols, including T-chol, LDL-c, and HDL-c, in patients with HIV infection [16, 30, 31]. As HDL-c and LDL-c have opposite effects on the development of atherosclerosis, the effects of changing both serum parameters are not clear. Recently, we reported that TDF for patients with HBV decreases serum cholesterol levels, including non-HDL and oxidized LDL, which are strongly associated with atherosclerosis [15, 18, 19, 28]. Macrophages phagocytize oxidized LDL-c and are subsequently transformed into foam cells, resulting in atherosclerosis plaque formation [32]. Blocking the uptake of oxidized LDL-c by macrophages inhibits the development of atherosclerosis [19]. Thus, the reductions in serum oxidized LDL-c and non-HDL levels by TDF are believed to reduce the risk of atherosclerosis. In our study, we showed that switching from TDF to TAF significantly increased levels of non-HDL-c and oxidized-LDL. Therefore, the risk of development of atherosclerosis might increase after a switch from TDF to TAF.

Both TDF and TAF are pro-drugs of tenofovir [8], and the mechanisms by which switching from TDF to TAF increases non-HDL-c and oxidized-LDL levels are therefore unclear. The difference in the dosage of TAF (25 mg once a day) and TDF (300 mg once a day) might explain the observed differential effect on cholesterol levels. The lower bone and kidney toxicity of TAF has been attributed to the lower required dosage of TAF (25 mg once a day) than that of TDF (300 mg once a day) [8].

We recently evaluated the mechanisms underlying the effect of TDF on cholesterol levels [15]. TDF reduced supernatant cholesterol in hepatocytes through the activation of PPAR-α-mediated signaling in vitro. The activated PPAR-α-mediated signaling upregulated PPAR-α target genes, including *CPT1* and *CD36* [33]. Hepatic CD36 uptook free fatty acids [34], oxidized LDL-c [35], and HDL-c [36]. Furthermore, we revealed that the effect of TDF in reducing supernatant cholesterol was dose-dependent; therefore, the attenuation of the effect on cholesterol levels might be related to the difference in required dosage between TAF and TDF.

Moreover, the rate of dyslipidemia, according to LDL-c and T-chol levels, as defined by the NCEP-ATP III lipid risk classification, increased after switching from TDF to TAF (from 0% with TDF administration to 14.4% for T-chol and from 13.0% to 31.4% for LDL-c). The rate of dyslipidemia determined by HDL-c levels decreased (from 23.2% with TDF administration to 7.2%). Overall, the rate of dyslipidemia based on any cholesterol increased from 33% to 39% after switching from TDF to TAF. Additionally, after switching, 1.4% and 5.8% of patients showed severe dyslipidemia based on T-chol and LDL-c, respectively, which was not observed pre-TAF treatment. Based on these findings, switching from TDF to TAF could have negative effects on the lipid profile, both qualitatively and quantitatively. Thus, this switch should be considered with caution.

Increased cholesterol levels after switching from TDF to TAF might affect intra- and extrahepatic disorders in patients with HBV infection. A high T-chol level in men with HBV infection is associated with an increased risk of HCC [37]. Thus, in such patients, the increase in cholesterol levels by switching from TDF to TAF requires careful monitoring for HCC. Additionally, TDF reduces the incidence of heart failure, which is sometimes associated with atherosclerosis [38] and reduces the lower common carotid intima-media thickness in patients with HIV infection [39]. Thus, after switching from TDF to TAF, the incidence of atherosclerosis-related diseases also requires careful attention.

The limitations of our study need to be acknowledged. First, it was a retrospective study with a relatively limited sample size. Furthermore, we analyzed cholesterol levels in preserved serum samples, which were collected regardless of food intake, preventing accurate assessments of changes in serum triglyceride levels. Additionally, data related to physical activity and energy intake were lacking, which could affect lipid metabolism, and data of cholesterols that changed after TAF administration in nucleotide analogue-naive HBV patients were not obtained. Therefore, larger prospective studies are required to validate our results.

## Conclusion

To the best of our knowledge, we provide the first evidence that switching from TDF to TAF not only increases T-chol, LDL-c, and HDL-c levels but also increases oxidized LDL-c and non-HDL-c levels in patients with chronic hepatitis B. In addition, switching from TDF to TAF increased the proportion of patients with dyslipidemia. As oxidized LDL-c and non-HDL-c are strongly associated with atherosclerosis, these changes should be carefully assessed when switching from TDF to TAF.

## Supporting information

S1 Fig Changes in cholesterols after switching from TDF to TAF in patients with chronic hepatitis or liver cirrhosis. A. Changes in T-chol, HDL-c, LDL-c, non-HDL-c, and oxidized LDL-c from pre-TAF to post-TAF administration (6–12 months) in patients with chronic hepatitis; n = 55. B. Changes in T-chol, HDL-c, LDL-c, non-HDL-c, and oxidized LDL-c from pre-TAF to post-TAF administration (6–12 months) in patients with chronic hepatitis; n = 14. TDF, tenofovir-disoproxil-fumarate; TAF, tenofovir alafenamide; T-chol, total cholesterol; HDL-c, high-density lipoprotein cholesterol; LDL-c, low-density lipoprotein cholesterol. Data are shown as means ± standard deviation for triplicate assays. *P < 0.05 or **P < 0.01.
(TIF)
