# Supplementary figures and images for: Effect of switching from tenofovir disoproxil fumarate to tenofovir alafenamide on lipid profiles in patients with hepatitis B
Source: PLoS One. 2022 Jan 20;17(1):e0261760. doi: 10.1371/journal.pone.0261760 (PMC8775237; doi:10.1371/journal.pone.0261760)

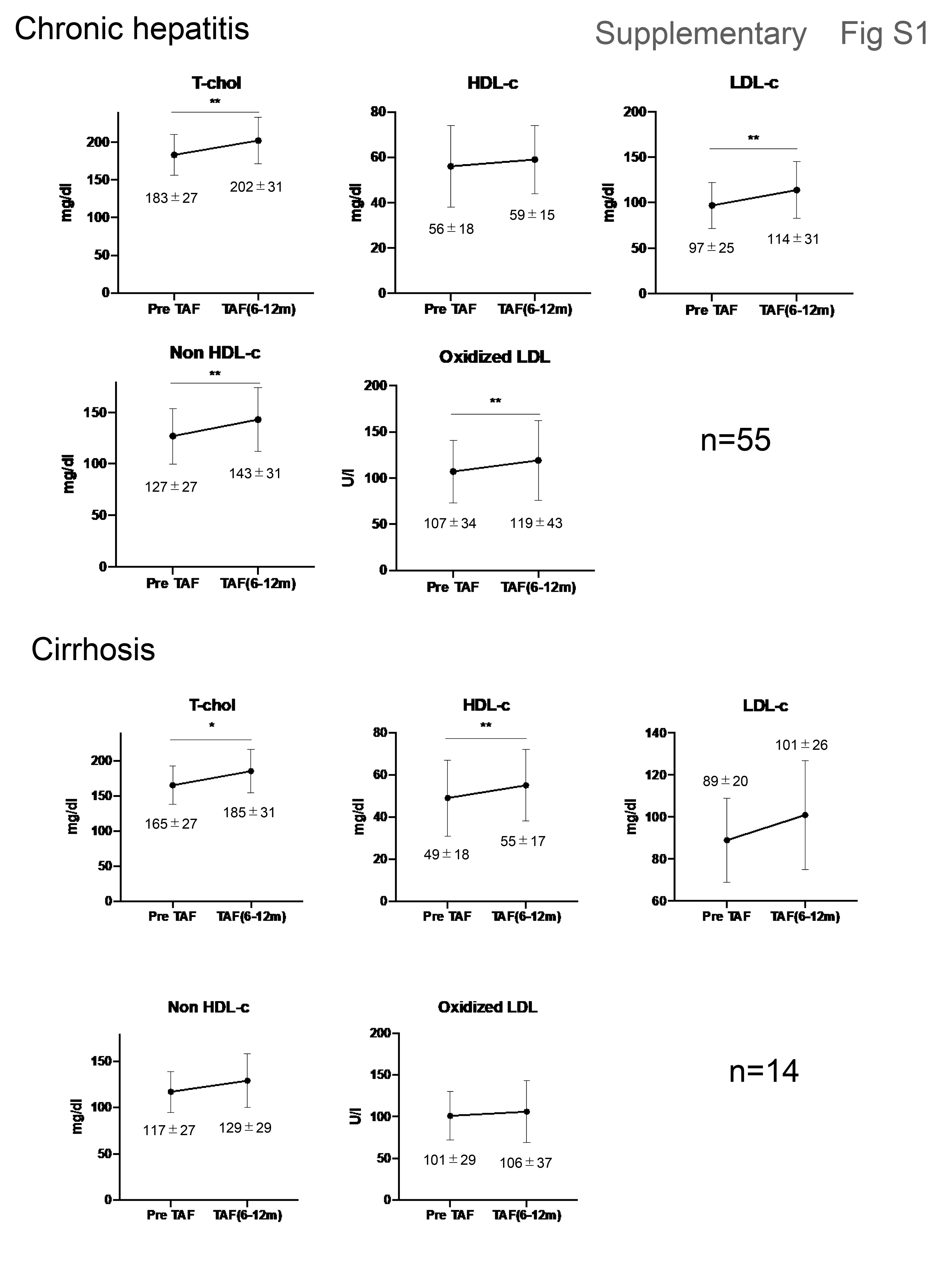

Supplement: S1 Fig — A. Changes in T-chol, HDL-c, LDL-c, non-HDL-c, and oxidized LDL-c from pre-TAF to post-TAF administration (6–12 months) in patients with chronic hepatitis; n = 55. B. Changes in T-chol, HDL-c, LDL-c, non-HDL-c, and oxidized LDL-c from pre-TAF to post-TAF administration (6–12 months) in patients with chronic hepatitis; n = 14. TDF, tenofovir-disoproxil-fumarate; TAF, tenofovir alafenamide; T-chol, total cholesterol; HDL-c, high-density lipoprotein cholesterol; LDL-c, low-density lipoprotein cholesterol. Data are shown as means ± standard deviation for triplicate assays. *P < 0.05 or **P < 0.01. (TIF) [file pone.0261760.s001.tif]
